# Partial culling and HPAI control in poultry: insights from Lower Saxony, Germany

**DOI:** 10.3389/fvets.2026.1787732

**Published:** 2026-03-19

**Authors:** Martin J. Oettler, Katja Schulz, Susanne W. F. Eisenberg, Christiane Mamarvar, Torsten Jäger, Johanna Rautmann, Carola Sauter-Louis, Ursula Gerdes

**Affiliations:** 1 Institute of Epidemiology, Friedrich-Loeffler-Institut, Federal Research Institute for Animal Health, Greifswald-Insel Riems, Germany; 2 Niedersächsische Tierseuchenkasse (Animal Disease Fund of Lower Saxony), Hannover, Germany; 3 Veterinary and Food Inspection Authority, Nienburg, Germany; 4 Office for Migration, Order and Consumer Protection: Veterinary Services and Animal Welfare, Winsen, Germany; 5 District Veterinary Authority, Cuxhaven, Germany

**Keywords:** avian influenza, biosecurity, epidemiological unit, epidemiology, poultry, segmentation

## Abstract

Outbreaks of highly pathogenic avian influenza (HPAI) pose a significant threat to the poultry industry. Enormous numbers of poultry are destroyed annually, not only by the disease itself, but also related to the control measures. One way to reduce these losses could be to apply partial culling methods to holdings where population destruction is mandated. This case series presents three cases from Lower Saxony, Germany, from 2021 or 2023, in which partial culling initiatives were successfully implemented. The cases include a turkey rearing farm with coupled parent stock, a free-range goose pasture for breeding, and a turkey fattening farm. Throughout the individual risk assessment process, the possibility of partial culling was recognized in all three cases. These measures saved approximately 57,000 animals, enabling them to fulfill their original purpose or provide secondary benefits. However, it should be noted that there were also unforeseen consequences during or after the partial culling approach in each case. The successful application in the individual outbreaks demonstrates that partial culling can be a suitable approach in specific situations. However, very high biosecurity standards and other necessary precautions are required, as well as a specific risk assessment focusing specifically on this option.

## 1 Introduction

Highly pathogenic avian influenza (HPAI) is a panzootic disease that occurs worldwide. In the European Union, it is categorized as a category A disease, for which immediate eradication measures must be taken (1, 2). These measures almost always involve the culling of all susceptible animals within the affected poultry holding during an outbreak. This often results in severe losses for individual farmers. Dozens of millions of poultry are destroyed annually during control efforts (3–5). A single country’s economic losses associated with this animal disease can reach billions of dollars per year (6, 7).

In the incident of an outbreak of HPAI in a poultry holding, the local veterinary authority must quickly decide how to handle the situation. The standard procedure for dealing with an outbreak is to destroy the entire population of poultry in the affected holding. However, if the animals are kept in separate epidemiological units, there are alternatives to culling the entire flock. According to Article 13 of Commission Delegated Regulation (EU) 2020/687, a derogation from the entire livestock culling approach is possible (8). Thus, a partial culling approach can be applied to the infected epidemiological unit only. This approach seems to create a higher level of social acceptability within the society and offers significant benefits from the perspectives of sustainability and animal welfare (9).

**Figure 1:**
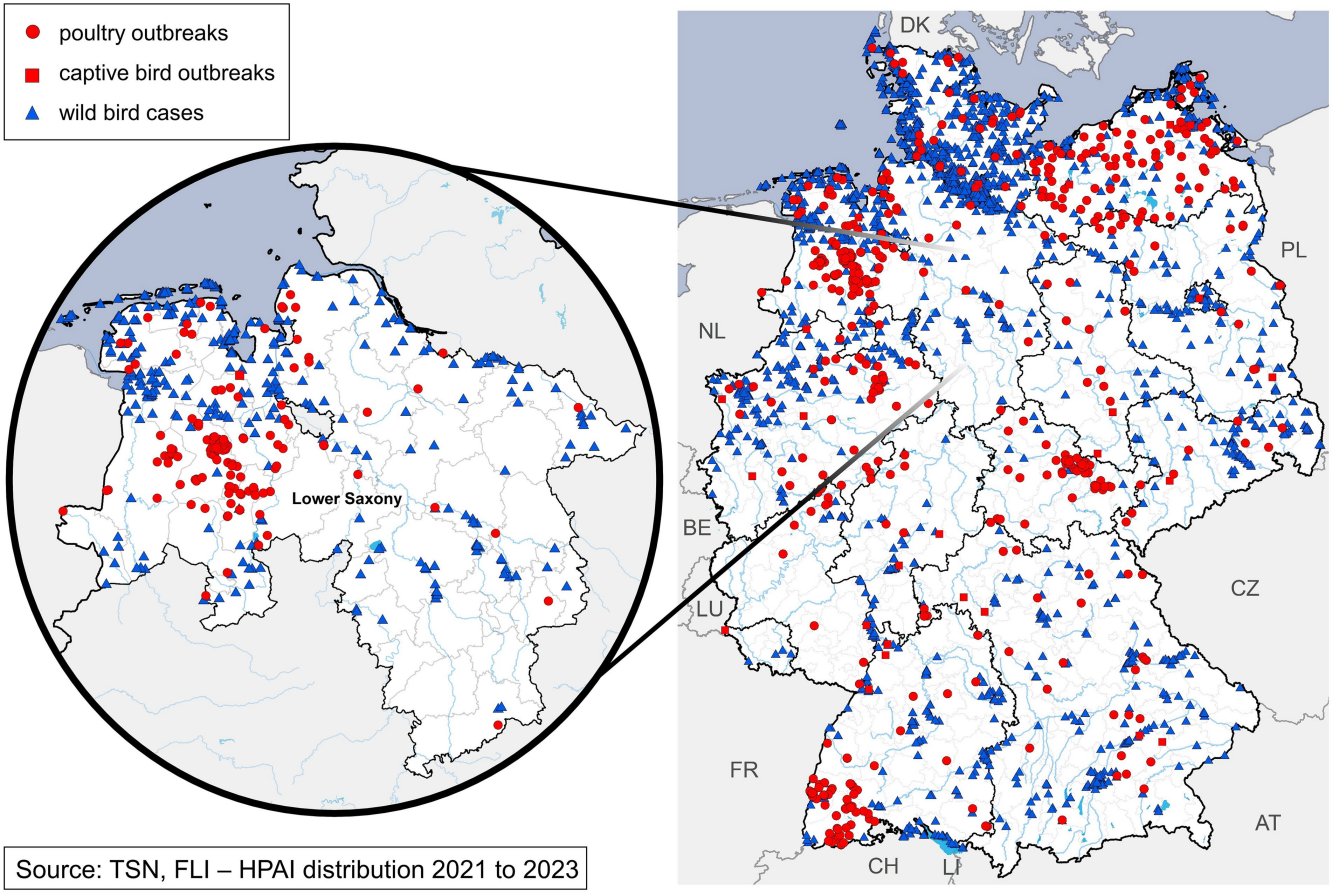
Distribution of outbreaks and cases of HPAI 2021–2023 in Germany with closer look for the federal state of Lower Saxony.

The federal state of Lower Saxony is the hub of German poultry production. Over 85 million poultry, accounting for 50% of the total poultry stock in Germany, are located here, which is more than the human population of the entire country (10). From 2021 to 2023, Lower Saxony reported 147 outbreaks of HPAI in poultry or captive bird holdings and 553 cases of HPAI in wild birds (Figure 1) (11).

This case series is a follow-up to a previously published paper in which a partial culling method was reported under “best practice circumstances” regarding biosecurity (12). However, as best practice examples are rare in the field, the following cases aim to describe the partial culling method in more common circumstances, as well as discussing the unforeseen consequences that occurred in relation to the partial culling approach. The subsequent cases are all examples of HPAI outbreaks in the federal state of Lower Saxony and are intended to provide a deeper insight into the animal disease outbreaks and the decision-making process of the responsible competent authorities regarding the partial culling approach. The base data for each individual case report came directly from the official district veterinarians, who were directly involved in outbreak management. Each case report is structured as follows: general information; outbreak description; risk assessment; culling method; subsequent requirements; and most likely path of infection. These cases may serve as examples in similar situations.

## 2 Case presentations

### 2.1 Case 1: Turkey rearing farm with coupled parent stock

The case occurred in late fall of 2021. The affected turkey farm consisted of three barns and housed approximately 9,900 turkeys for rearing, which were 21 weeks old at the time of the outbreak. Another turkey farm with five barns, which housed breeder turkeys for egg production was located half a kilometer away. This neighboring farm had a total of approximately 8,800 birds. Both farms were owned by the same company, but they operated independently with separate national registration numbers. However, as it had been verified that staff were operating on both farms in recent days, the laying farm was considered a contact farm in relation to the outbreak.

Due to increased mortality (seven deceased animals) and clinical signs in one barn, the veterinarian monitoring the flock performed sampling (oropharyngeal and cloacal swabs) and a clinical examination. The samples were tested in a private laboratory and found to be positive for avian influenza virus (AIV). The local veterinary authority was notified, and 25 official swab samples were collected from birds in the affected barn that same day. The Lower Saxony State Laboratory detected AIV H5 in all 25 samples. The National Reference Laboratory at the Friedrich-Loeffler-Institut confirmed the results by detecting highly pathogenic avian influenza virus (HPAIV) H5N1.

Because of the local authority’s risk assessment and the absence of clinical signs in the animals of the laying farm the local authority classified the two neighboring farms as different epidemiological units in accordance with Article 20 of Commission Delegated Regulation (EU) 2020/689 (13). Despite the fact that same workers operated on both farms, each farm had an individual hygiene sluice with a shower, farm-specific clothing with a washing machine, and a shoe change for each barn. Additionally, there were no shared devices between the farms. Overall, the farms had a very well-implemented biosecurity management system. The absence of other larger poultry farms in the vicinity was also a beneficial factor in the assessment. A critical aspect in the risk assessment was that the only transportation route for the carcasses from the outbreak farm passed directly between the barns with the parent animals. To reduce this risk, the trucks were disinfected directly on the premise of the rearing farm, so before passing the laying farm.

The animals in the three barns of the rearing farm were destroyed by fumigating the stables with carbon dioxide. Afterwards, cleaning, disinfection, and manure storage were carried out according to the veterinary authorities’ requirements. On the same day as the culling, a clinical examination was performed at the laying farm and swab samples were taken from four deceased birds, neither revealed evidence of the virus. Therefore, a derogation from the destruction of the parent birds was granted in accordance with Article 13 of Commission Delegated Regulation (EU) 2020/687 (8).

For surveillance purposes, the local veterinary authority conducted clinical examinations of all poultry farms within the 3 km protection zone and, based on risk, within the 10 km surveillance zone. Another turkey farm located 18 km away from the outbreak was sampled because it belonged to the same company as the outbreak farm. However, due to its distance and completely separate management system, it was not considered a contact farm. All samples were tested negative for AIV. Also, the samples from all deceased animals on the parent farm tested negative continuously until 2 weeks after the outbreak, when the turkeys were transported to a nearby (80 km) slaughterhouse. Prior to transport, the local veterinary authority took 20 official swab samples (oropharyngeal and cloacal) per barn section and tested them negative for AIV at the Lower Saxony State Laboratory. In accordance with Article 29 of Commission Delegated Regulation (EU) 2020/687, the local veterinary authority authorized the transport, supervised the loading, and sealed the truck (8).

The most likely way the virus entered was through the ventilation system. There was a pond with a wild bird population near the farm where the outbreak occurred. In the days before the outbreak, the wind vane was directed toward the barns’ ventilation openings. Entry by people, vehicles, or materials was considered unlikely.

### 2.2 Case 2: Free-range geese pasture for breeding

**Figure 2:**
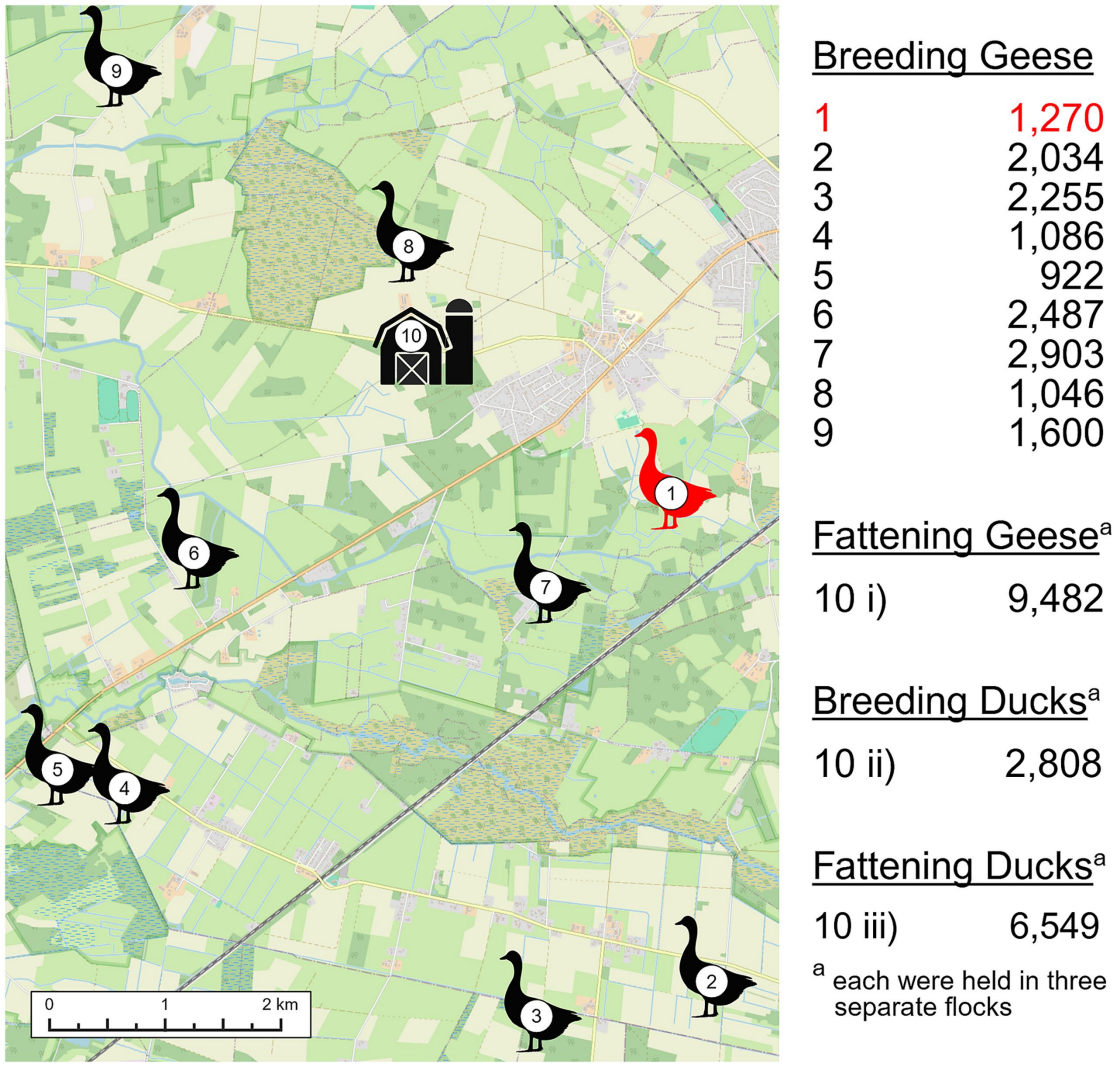
Distribution and number of geese held on pastures and ducks held in barns that were assessed as contact farms. The red goose symbolized the outbreak pasture. Map based on OpenStreetMap. Created in BioRender, Oettler, M. (2026) https://BioRender.com/8aokxwu.

The outbreak occurred in late fall/early winter of 2021. In the affected goose pasture, which was around 27,000 m^2^, 1,270 great-grandparent geese used for breeding were held. The geese were approximately 5 years old. The company had eight other free-range pastures for geese, three pastures for fattening geese, three flocks of breeding ducks in barns and three flocks of fattening ducks in barns (Figure 2). Each location had its own national registration number. However, due to person (footwear) and vehicle contact with biosecurity aspects in need of improvement, all flocks were considered contact flocks in relation to the outbreak.

At the end of November, the farmer responsible for the geese flock noticed abnormalities in their clinical behavior. Three days after these first signs appeared, the mortality amounted to 50 animals. The local veterinary authority was informed and took 20 official samples from the deceased geese. Upon observing the clinical signs, the slaughter of fattening animals at the company’s on-site slaughterhouse was halted, and the care of multiple flocks by a single staff member was prohibited immediately. The Lower Saxony State Laboratory detected AIV H5 in all 20 samples the same day, and the National Reference Laboratory at the Friedrich-Loeffler-Institut confirmed it as HPAIV H5N1 the day after.

In the risk assessment conducted by the competent authorities, all flocks were considered as one unit in terms of epidemiological and hygienic understanding. However, due to the significant time and equipment required for a culling process across several free-range pastures, a close-meshed sampling process (described in the following sections) was deemed a far more viable alternative. Another point raised in the assessment was that none of the geese or ducks in the contact pastures showed any clinical signs. However, due to the large number of contact pastures, the competent authority issued a general ruling for compulsory stabling across the entire district, in addition to setting up restriction zones. One pasture (Pasture 3 in Figure 2) was located just beyond the district boundary in another district. The local authority of the respective district immediately ordered a sampling of the geese in this pasture. This was in addition to a routine monitoring of the animals, that had recently taken place approximately 1 week prior.

The geese in the outbreak pasture were destroyed by container fumigation with a mixture of argon and carbon dioxide. Afterwards, cleaning and disinfection were carried out under the direction of the local authority. Furthermore, an exemption was granted for plowing permanent grassland after applying quicklime to the outbreak pasture. On the day of the culling, sampling began in all other pastures, with an initial sample size of 40 oropharyngeal swab samples per pasture, taken by the competent authority. The Lower Saxony State Laboratory tested all samples negative for AIV.

Following the initial sampling, the respective district authority set the sampling timeframe to thrice weekly for 3 weeks, with 20 oropharyngeal swabs sampled per flock. Additionally, every deceased animal was sampled. After three rounds of negative test results, the sampling was restricted to the fattening animals only. The negative test results were valid for 48 h, enabling continuous slaughter of fattening animals, with 560 geese and 390 ducks per day, at the company’s on-site slaughterhouse. Throughout the entire process, the State Laboratory of Lower Saxony tested all samples negative for AIV. The breeding geese were continuously housed in barns or field tents. However, due to the large number of animals, implementing housing to all the pastures took until shortly after the turn of the year. An application for an exemption permit to the compulsory housing was rejected by the authorities. Additionally, biosecurity measures were significantly improved for all flocks. Hygiene sluices with disposable clothing were installed, and each flock was managed by a different person. The herding dogs from each pasture were also cleaned properly and kept away from the poultry flocks. In addition, the veterinary authority requested reporting of the number of deceased birds from the 340 poultry holdings located within the protection and surveillance zones. Furthermore, a risk-based clinical examination of the poultry holdings in the protection zone was conducted, along with a short epidemiological survey.

The HPAI infection of the geese in the pasture where the outbreak occurred, very likely happened via direct contact with wild birds or their fomites. The timing of the outbreak coincided with the wild bird migration, and other cases of HPAI were reported in wild birds nearby.

### 2.3 Case 3: Two Turkey holdings for fattening

The HAPI outbreak occurred at the end of winter in the beginning of the year 2023 on a fattening farm for turkeys. The farm held around 15,000 male turkeys, which were located in four barns. The gobblers were 17 weeks old and weighted around 18 kg. Another turkey farm, for fattening and combined rearing and fattening, which housed also around 15,000 male turkeys in two barns, was located 1 km away. Although both farms belonged to the same family-owned company, they operated independently and had their own national registration numbers. However, since staff and the veterinarian had operated on both farms in the recent days before the outbreak, the second farm was considered a contact farm in relation to the outbreak.

During a site visit, the veterinarian monitoring the flock noticed some deceased animals and altered clinical behavior in one of the barns of the fattening farm. He conducted a rapid test specific to AIV, which yielded a positive test result. He immediately informed the competent authority. This test result was confirmed by a positive PCR test from a private laboratory, afterwards. On the same day, the veterinary authority took 20 official swab samples (oropharyngeal and cloacal) from the gobblers of each barn, as well as 20 official swab samples per barn from the animals in the contact farm. The Lower Saxony State Laboratory detected AIV H5 in the majority of samples from the barn where the animals exhibited clinical signs. These samples were sent to the National Reference Laboratory at the Friedrich-Loeffler-Institut, which confirmed the result by the detection of HPAIV H5N1.

The next day, clear clinical signs such as elevated mortality, a sharp decline in feed intake, and central nervous system disorders were evident in the infected barn. Due to the absence of these signs in the contact farm, the negative AIV test results of the animals in its barns, and the biosecurity protocols in place, the competent veterinary authority classified the two farms as separate entities in epidemiological understanding. A critical aspect of the risk assessment was that staff sometimes operated on both farms, but this practice was immediately ceased after the first signs of the disease appeared in the first flock. Overall, the biosecurity measures on both farms were considered good since they had recently been improved following an HPAI outbreak at the same establishment approximately 2 years prior. Standards such as the use of hygiene sluices with separate black-and-white areas for the changing into farm-specific clothing and footwear were followed, and the owner was in detail sensitized to the importance of these biosecurity measures.

All of the gobblers in the four barns on the outbreak farm were destroyed through stable fumigation with carbon dioxide. The next day, the carcasses were brought to the rendering plant. Afterwards, cleaning, disinfection, and manure storage were carried out. These were done according to the veterinary authorities’ requirements. On the same day as the carcass disposal, testing of deceased birds on the contact farm began. Since the results for AIV were negative, no animals of the contact farm were destroyed. Epidemiological samples (60 swabs per barn) were taken during culling and analyzed at the Lower Saxony State Laboratory. All samples from the barn with gobblers displaying clinical signs tested positive. For the two direct neighboring barns, the prevalence was 75% (95% CI: 62.1–85.3) and 6.7% (95% CI: 1.8–16.2), respectively, recent virus transmission was therefore plausible. The samples from the animals in the last barn revealed no virus presence.

To establish an effective surveillance system, the respective district veterinary authority mandated a sampling protocol for all deceased birds on the contact farm, conducted twice weekly. To enhance this system, the sampling scheme was extended to include all commercial poultry holdings within the 3 km protection zone. This was combined with a visit and clinical examination of all 42 poultry holdings in the zone. For the 235 poultry farms within the 10 km surveillance zone, the veterinary authority conducted a visit to all farms with more than 300 birds or those who held turkeys. Throughout the entire process, no clinical signs were observed, and all samples taken from deceased birds tested negative for AIV at the State Laboratory of Lower Saxony. After the outbreak was successfully managed and the restriction period was over, the farm owner was allowed to bring new turkeys, provided that a virological examination via PCR was conducted on 60 birds per barn prior to transportation and again between weeks two and three after transfer. These samples also tested negative.

Regarding the most likely path of infection, it was assumed that the directly adjacent wild waterfowl population of the North Sea played a decisive epidemiological role. It was considered unlikely that the virus entered via people, material, feed, or bedding.

## 3 Discussion

The application of partial culling methods following an outbreak of an infectious animal disease is not quite common. This possibility is set forth in EU legislation, specifically in Article 13 of Commission Delegated Regulation (EU) 2020/687 (8). However, the respective local authority is responsible for its implementation. This approach requires more effort than the “standard” approach of complete culling, especially for the subsequent sampling. Furthermore, if the approach fails, the respective authority will be questioned in much more detail about their reasoning.

However, the application of a partial culling procedure is an excellent example of how to fulfill the three pillars of sustainability (14). From the social perspective, the destruction of contact animals is very difficult for society to comprehend and ethically to justify (9, 15). An open communication strategy, explaining the partial culling approach could create more appreciation for combating animal diseases. It would also increase understanding of which animals must be compulsorily destroyed for epidemiological and animal welfare reasons. Also, from an economic standpoint, a partial culling approach could give an occasion to partly reduce the severe monetary losses in terms of fighting an animal disease. These high loses are often associated with the loss of genetics of the animals. If a flock of highly valuable parent or grandparent animals is affected by an animal disease, the value of each animal increases significantly because the loss of these animals could have a domino effect on the subsequent production processes. The last pillar concerns the environmental benefits. Specifically, any healthy livestock animal fulfilling its intended purpose can be considered beneficial with respect to greenhouse gas emissions, as its absence would necessitate replacement by another animal’s production, to compensate the loss of the output, and therefore create additional emissions (16).

Partial culling is not often undertaken in practice. In livestock production, it was recently considered for use in African swine fever control (17). In the poultry sector, only a few cases have been reported in best practice example’s, zoos or wild bird rescue centers (12, 18, 19). Reasons that these control measures are uncommon in the poultry sector may be due to the additional precautions and subsequent efforts that are required. Also, in all three of our reported cases, unforeseen consequences occurred during, after or in relation with the partial culling approach:

In Case 1, the slaughter of the parent animals was not intended as the final solution for the turkeys. Since these were parent animals, they produced breeding eggs. The pickup and delivery of these eggs was paused on the day of the positive AIV test result from the private laboratory. After the initial outbreak farm was destroyed, the farmer asked the local authority for permission to move the eggs out of the protection zone to the hatchery. The local authority granted this in accordance with Articles 31, 34, 47, and 50 of Commission Delegated Regulation (EU) 2020/687 (8). However, the hatchery denied taking the eggs because, among other reasons, they were afraid of a trade embargo since they actively participated in European commercial intercourse with day-old chicks. Potentially infected eggs would therefore be a catastrophic event with far-reaching consequences. The farm owner’s attempts to sell the hatching eggs to other trading partners were also unsuccessful, which put enormous financial and emotional pressure on the farmer (20). Consequently, the eggs were disposed of as a category 3 animal by-product within a biogas plant in accordance with Article 37 of Commission Delegated Regulation (EU) 2020/687 (8). Since the initial purpose of the animals could no longer be fulfilled, the farmer decided to send them to slaughter.

In Case 2, the most important point was that culling of animals on all pastures at different locations would have taken far too long. The risk assessment assumed a 10 day period to destroy all birds belonging to the company where the initial outbreak occurred. Also, the lack of electricity and water supply close to the free-range herds would have complicated the process and increased costs massively. Therefore, the sampling method was an alternative much quicker, and the fact that no further infections occurred, confirmed it was the right decision. Another reason for partial culling was that the breeding animals were great-grandparent animals, meaning they had highly valuable genetics and were of great interest to save. The rapid slaughter of the fattening birds at the company’s on-site approved slaughterhouse following the outbreak was associated with the pre-Christmas period and the high demand for goose, duck and turkey meat (21, 22). Transporting and slaughtering the birds within the protection zone, as well as transporting the carcasses out of the protection zone, was permitted in accordance with Article 29 and 33 of Commission Delegated Regulation (EU) 2020/687 (8).

For Case 3, it should be noted that the partial culling approach is not always successful. Specifically, another primary outbreak occurred at the afore-named contact farm at the beginning of 2021 (HPAIV H5N8), and also at the aforementioned outbreak farm at the end of 2023 (HPAIV H5N1). In both cases, the partial culling approach was tried for the respective contact farm, but later rejected when a secondary outbreak occurred in these. A surveillance system focusing on deceased animals at the contact farm was set up in both cases and indicated an outbreak after 4 or 6 days, respectively, which was within the likely incubation period of the virus. Due to direct person contact, it is likely that the virus entered before the primary outbreak was noticed and the staff managing both farms were segregated. Nevertheless, the secondary outbreaks could be handled, and the animals in the respective barns were destroyed. These approaches demonstrated that the partial culling approach can fail, e.g., if the epidemiological segmentation is violated due to person contact with inadequate biosecurity standards. But, more importantly, the partial culling approach can only work if it is actively implemented in the practice, even when circumstances do not always align with the “best practice” example. Evidence of this was shown in the case of early 2023, where partial culling was successful.

## 4 Conclusion

All three cases demonstrated that partial culling initiatives can work, if a thorough risk assessment outlines this possibility. The application of the partial culling approach in all three cases were considered successful, as the spread of the disease stopped while the animals on the respective contact farms remained unaffected and were not destroyed. In total, around 57,000 animals were saved by this approach and could be used for their intended purpose, or for secondary benefits in meat production. These cases also showed that, even when field situations do not align with the highest biosecurity standards prior to an outbreak, a partial culling approach can be viable and worth trying. This is because avoiding a full culling approach has great benefits in terms of the economy, sustainability, and animal welfare. However, it must be acknowledged that there are consequences for the society that must be accepted, such as consuming meat originating within the protection zone or that the meat products can be only sold pre-cooked for safety reasons. In terms of poultry holdings, a partial culling initiative is beneficial for saving animals. However, implementing separate epidemiological units based on solid biosecurity standards as a precaution to prevent animal disease outbreaks is an even better strategy. This would help to avoid unforeseen consequences and also promote animal welfare.

## Data Availability

The raw data supporting the conclusions of this article will be made available by the authors, without undue reservation.
